# Notes on Current Topics

**Published:** 1909-02-13

**Authors:** 


### Motoring Notes.

### NOTES ON CURRENT TOPICS.

### Cape Cart Hoods.

In regard to the subject of Cape hoods, which was recently alluded to in these notes, I have since read in the *Autocar* some rules for their use, which are so amusing and yet so practical as to be worthy of reproduction. They are as follows: "Never furl a hood until it is dry. If lady passengers insist on the hood being refurled when a shower is over, resist them to the death, if wife or blood relation; if guest or *fiancée*, submit, but put your hood up again to dry on reaching the garage unless it be made of Kamac, which is the only material we have found which can be furled wet without damage." The experience of the *Autocar* with Kamac is thus similar to my own as the only material used for Cape hood covering that will effectually resist damp and also retain its colour and smart appearance.

Whilst writing of Cape hoods I would say that in ordering a new hood it is necessary to see that the tilt comes well forward over the bonnet, or else there is poor protection for the driver. If a wind screen is used in conjunction, and if there are any gaps between the top of the screen and the roof of the hood, have a short drop curtain made to cover it. Side curtains are very necessary in wet weather, especially with driving rain; but it is essential that they should be well fitted or they will flap in windy weather.

### An Efficient Silencer.

Last autumn Messrs. J. C. Lyell and Co., of 55 Victoria Street, Westminster, asked me to try their "Clair" Silencer, for which they claimed a degree of efficiency hitherto unattainable without loss of power through back pressure. Having tried various makes of silencers, for which many virtues were claimed by their inventors, it was with considerable diffidence that I agreed. For testing purposes I chose a 6 h.-p. single-cylinder car very heavy on the exhaust, with whose running power I was exceptionally familiar. I was considerably surprised with the result, as the exhaust was practically inaudible when the car was running with the engine at all throttled, and I could only hear the noise of the automatic inlet-valve. Hitherto the exhaust completely drowned all other sources of noise. I also found a total absence of back pressure; in fact, the engine gave more power and I was able to run up hills on top speed which formerly I frequently had to negotiate by changing down. This demonstrates the remarkable difference in the running of a small car that can be attributed to silencer design. The "Clair" Silencer is, like many efficient inventions, of simple design. The escape of the gas is direct, and there is a total absence of all complicated parts, such as metal or asbestos gills, balls, etc., so that there is no chance of back pressure or fouling. It has no chamber where unconsumed gas caused by misfiring can accumulate and consequently all likelihood of explosion in the silencer is eliminated. It can be easily taken down for cleaning purposes; but, unlike most of its fellows, this will only be necessary at very long intervals. It obtained the highest award at the last International Trials, 1907, and the gold medal at the Travel Exhibition of the same year. It is not expensive, the price, of course, varying according to horse-power of engine, etc.; but for the average doctor's car the cost would be about £2 10s.

### The Passing of the Security Bolt.

Every car owner must at times have had cause to regret the necessity of security bolts, a frequent cause of tyre troubles, not to speak of loss of time and temper. Everyone who motors is familiar with the bolt which, after the cover is replaced in the rim, refuses to bed down in its place and necessitates the removal and replacement of the cover. This type is bad, but there is a worse, namely, the bolt that gives no sign of not being in its proper place until at some awkward time it gives evidence of the fact by a most startling report. The Michelin Company have recently brought out an addition to the valve stem which, they claim, abolishes the security bolt for ever. This takes the form of a boat-shaped plate with overturned, rounded edges and flattened tongued ends through which the valve tube passes, and in which it is secured by a thin hexagon nut. The valve is introduced into the rim within the cover in the usual way, so that the rounded edges of the plate rest on the beaded edges of the cover. When the ordinary milled nut is screwed down on the wheel felloe as usual, the cover is securely fastened in the rim and prevented from creeping without the aid of any security bolts. Many people long accustomed to consider a tyre insecure without bolts may feel doubtful whether the valve plate will prove efficient. The fact, however, that Messrs. Michelin are putting it on the market should be, to most motorists, sufficient guarantee of its effectiveness. They have not hitherto offered anything that they have not thoroughly tested. Having thus abolished security bolts and their numerous imperfections and troubles, they will gain the everlasting gratitude of all practical motorists.

As several gentlemen have recently written asking for my advice in regard to motor cars, I may say that I shall be pleased to reply to any letters sent to me care of the Editor. "Viator."

